# The National Medical Guild

**Published:** 1913-12-20

**Authors:** 


### THE NATIONAL MEDICAL GUILD.

The National Medical Guild will hold a meeting of medical practitioners on Friday, December 19, at 4 p.m., at the Hammersmith Town Hall, Hammersmith Broadway, for the purpose of a discussion on the value of trade unionism for the medical profession. The chair will be taken by Dr. Charles Buttar, President of the Guild. Mr. E. B. Turner, F.R.C.S., will open the discussion in favour of a trade union. Speeches and questions from the audience will be invited, and a resolution will be put to the meeting at 6 o'clock.

